# Cholesterol efflux responds to viral load and CD4 counts in HIV+ patients and is dampened in HIV exposed

**DOI:** 10.1194/jlr.M088153

**Published:** 2018-09-13

**Authors:** Olivia Tort, Tuixent Escribà, Lander Egaña-Gorroño, Elisa de Lazzari, Montserrat Cofan, Emma Fernandez, José Maria Gatell, Esteban Martinez, Felipe Garcia, Mireia Arnedo

**Affiliations:** Group of Genomics and Pharmacogenomics,* Acquired Immune Deficiency Syndrome Research Group, Catalan Project for the Development of a Human Immunodeficiency Virus Vaccine (HIVACAT), Institut d’Investigacions Biomèdiques August Pi i Sunyer (IDIBAPS), Hospital Clinic de Barcelona, Barcelona, Spain; Infectious Diseases Service,† Hospital Clínic-IDIBAPS, Laboratory of Retrovirology and Viral Immunopathogenesis, University of Barcelona, Barcelona, Spain; Lipid Clinic,§ Department of Endocrinology and Nutrition, Hospital Clínic de Barcelona, IDIBAPS, Barcelona, Spain and CIBER de Fisiopatología de la Obesidad y Nutrición (CIBEROBN), Instituto de Salud Carlos III (ISCIII), Madrid, Spain; Hospital Clinic/IDIBAPS,** University of Barcelona, ViiV Healthcare, Barcelona, Spain

**Keywords:** CD4+ T-cell, human immunodeficiency virus-infected, human immunodeficiency virus-exposed seronegative, high density lipoprotein cholesterol, apolipoprotein AI, cardiovascular disease, lipoprotein (a)

## Abstract

Cholesterol efflux (CE) capacity has been inversely associated with atherosclerosis and may provide an insight on inflammation occurring in human immunodeficiency virus (HIV) individuals. We address this by studying CE in HIV patients at different stages of HIV disease progression. In this cross-sectional study, CE from ApoB-depleted plasma, lipids levels, viral load (VL), CD4+/CD8+ T-cells, high-sensitive C-reactive protein (hsCRP), and lipoprotein (a) were evaluated in untreated HIV-infected patients (UHIVs; n = 43), elite controllers (ECs; n = 8), HIV-exposed seronegative individuals (HESNs; n = 32), and healthy controls (HCs; n = 14). Among UHIVs, those with CD4+ <500 cells/mm^3^ presented the lowest significant CE, HDL cholesterol (HDL-C), and ApoAI levels. ECs showed similar HDL-C, ApoAI, and CE compared with HCs. Among UHIVs, CE positively correlated with CD4+ T-cell counts (Beta: 1.05; 95% CI: 1.02; 1.07), and for VL higher than 3.8 log, CE was inversely associated with VL (Beta: 0.70; 95% CI: 0.51; 0.95). Remarkably, HESNs presented higher CE (0.78 ± 0.14) than UHIVs (0.65 ± 0.17; *P* = 0.0005), but lower than HCs (0.90 ± 0.13; *P* = 0.009). hsCRP levels were highest in the UHIV group (0.45 ± 0.49). CE was sensitive to HIV disease progression. Low CE in HIV patients was associated with lower CD4+ T-cells and higher VL and hsCRP. CE was also lower in HESNs compared with HCs. Our results suggest that immune status secondary to HIV progression and exposure influence plasma HDL-CE capacity.

Plasma HDL functionality can be measured using a cholesterol efflux (CE) capacity assay, an emerging cell-based assay that successfully predicts cardiovascular events in the general population and could be used as a biomarker of atherosclerosis (1–3).

Previous reports have shown the impact of human immunodeficiency virus (HIV) infection on atherosclerosis as an inflammatory process influencing plasma lipids, endothelial dysfunction, and immune activation (4, 5). Several critical steps in the replication cycle of HIV are dependent on cholesterol (6–8), and it is also known that untreated HIV-1-infected patients present altered lipoprotein profiles in blood (9). HIV affects cholesterol homeostasis by downregulating ABCA1 (10), a transporter protein responsible for the major part of CE to HDL in macrophage foam cells (11). As a consequence, CE is decreased and it accumulates in the infected macrophages (12) leading to foam cell formation and atherosclerotic plaque progression (13, 14).

In the field of HIV, the CE capacity of plasma samples has been measured in few studies. Patients with uncontrolled HIV infection revealed impaired CE compared with healthy individuals, independently from HDL cholesterol (HDL-C) levels (15, 16), but no differences in CE were found in a similar setting (17). Antiretroviral therapy (ART) can restore CE to levels similar to those of healthy individuals (15, 18). Therefore, while the improvement of CE by ART is clear, the role of CE in viral replication on HIV infection needs to be clarified.

This study was designed to evaluate lipid fractions, lipoproteins, and cholesterol-associated parameters in patients at different stages linked to HIV infection. The groups included in the study were untreated HIV-infected patients (UHIVs), elite controllers (ECs), HIV-exposed seronegative individuals (HESNs), and a group of healthy controls (HCs). We hypothesize that not only the quantitative levels of HDL-C, LDL cholesterol (LDL-C), and ApoAI are altered but there may also be additional changes caused by HIV infection in the HDL particles, particularly in HDL functionality. We explore this by means of a CE assay optimized in THP1 cells (15, 19) and validated in our laboratory. We further evaluated the link of CE to the inflammatory marker, high-sensitive C-reactive protein (hsCRP), lipoprotein (a) [Lp(a)], and immunology data of the patients.

## MATERIALS AND METHODS

### Patients

Samples were obtained from HIV-1-infected patients followed-up at the HIV Unit-Hospital Clinic de Barcelona. Samples of uninfected donors were obtained from the Blood and Tissue Bank (BST, Barcelona, Spain). Ethical committee approval and written informed consent from all subjects were obtained prior to study initiation. The study was approved by the institution ethical committee: Comitè Ètic d’Investigació Clínica, Hospital Clinic, Barcelona, Spain (protocol approval number: HCB/2014/0756).

Ninety-seven individuals participated in this cross-sectional study and were classified into five groups according to their characteristics linked to HIV-infection: UHIVs CD4 <500 (n = 27; CD4+ T-cell count <500 cells/mm^3^, ART-naïve); UHIVs CD4 >500 (n = 16; CD4+ T-cell count >500 cells/mm^3^, ART-naïve); ECs [n = 8; viral load (VL) <50 copies/ml and CD4+ T-cell count >500 cells/mm^3^ for >6 years in the absence of ART]; HESNs (n = 32; seronegative at the time of inclusion and after 3 and 6 months of follow-up; with history of ongoing unprotected sexual intercourse in the past 3 months, with five episodes of unprotected sexual intercourse in the past 3 months, and with ≥1 episodes in the 4 weeks prior to study entry with the respective HIV-1-infected partner with a plasma VL >3,000 copies/ml); and HCs (n = 14). Individuals with diabetes and/or hepatitis C virus coinfection were excluded from the study. Consumption of lipid-lowering agents was reported.

The HIV-infected (HIV+) partners of the HESNs (n = 32; partners of HESNs and VL >3,000 copies/ml) are included in the UHIV groups described above. The HIV+ permitted a pairwise comparison of the HESN individuals to their HIV+ partners.

### Metabolic and inflammatory parameters

EDTA-plasma aliquots preserved at −80°C were used to determine total cholesterol (TC), HDL-C, LDL-C, triglycerides, ApoAI, ApoB, Lp(a), and hsCRP at the Biomedical Diagnostic Center of the Hospital Clinic Barcelona (Barcelona, Spain).

### ApoB-depleted plasma

ApoB-depleted plasma (ABDP) was obtained by removing LDL particles from plasma samples stored at −80°C as described by Asztalos et al. (20) with slight modifications. Briefly, previously thawed plasma was treated with polyethylene glycol (PEG) (PEG8000; Sigma, #202452-250G) solution [20% PEG in 200 mM glycine buffer (pH 7.4)] on ice (1:4). After 20 min, the precipitate was removed by centrifugation (13,000 *g*; 15 min at 4°C) to obtain the ABDP supernatant containing the HDL-enriched lipoprotein fraction. Note that the components of the final ABDP are 40% diluted from the original plasma sample.

### CE assay

The CE assay was successfully optimized using previously PMA-differentiated THP-1 cells and ABDP as cholesterol acceptor (supplemental Fig. S1). In order to increase throughput and simplify cell treatments, CE capacity from ABDP was quantified in triplicate using a slightly modified method described by Khera et al. (3) (see supplemental material for details on the CE assay description).

The average coefficients of variation (CVs) remained excellent along the duration of the study, with a CV of 5.5% between triplicates and a CV of 6.9% inter-batch (supplemental Table SI; see supplemental material for details on the validation of the method).

### Statistical analysis

Data are shown as mean and standard deviation or median and interquartile range (IQR; 25th to 75th percentile), unless otherwise specified. One-way ANOVA (parametric) or Kruskal-Wallis (nonparametric) analyses were used to compare variables between groups. Tukey’s (parametric) or Dunn’s multiple comparison test (nonparametric) were used as posttests. In order to compare parameters between two groups, *t*-test or Mann-Whitney were used. Paired *t*-test was used to determine statistical significance between means of HESNs and their HIV+ partners. Contingency tables of qualitative characteristics distribution between groups were compared with Fisher’s exact or χ^2^ test (GraphPad Prism version 5.00, San Diego CA).

The influence of CD4+ T-cell counts and HIV-RNA plasma VL adjusted for age on CE in UHIVs were estimated using ordinary least squares regression model. The CE was considered log-transformed to reduce skewness and improve residual distribution. Hence, the regression coefficient was exponentiated and represented the ratio of the (geometric) mean of CE corresponding to a 100 cell increment in CD4+ T-cells and to one log_10_ increase in plasma VL. Nonlinear form in VL was also tested and the quadratic specification was chosen, meaning that the size and sign of the VL effect on CE depend on the value of VL.

A regression of Ln-CE on CD4 and VL by using a piecewise linear function for log_10_ VL with a knot at 3.8 was also estimated (adjusted for age). In HESNs, the effect of CD4+ T-cells (percent) and CD8+ T-cells (percent) on the CE (percent) adjusted for age was estimated using the ordinary least squares regression model. Confidence level was set at 95%. Models were estimated using Stata (StataCorp.2013. Stata: Release 13 statistical software; StataCorp LP, College Station, TX).

## RESULTS

### Characteristics of the study participants

Characteristics of the study participants are shown in **Table 1**. Patients were overall 80% men, predominantly men who had sex with men. Sex and age distributions were similar between groups (Fisher’s exact test, *P* = 0.16; ANOVA one-way test, *P* = 0.68, respectively). UHIV patients were grouped according to their CD4+ T-cell count into UHIV CD4 <500 and UHIV CD4 >500. As expected, the UHIV CD4 <500 group presented higher levels of plasma VL than the UHIV CD4 >500 group (Mann Whitney test; *P* < 0.0001). CD4+ T-cell (expressed in cell count and in cell percentage) differed between groups (ANOVA one-way test; *P* < 0.0001 in both cases). CD8+ T-cell count was similar between groups (ANOVA one-way test; *P* = 0.249; nonsignificant), while it differed significantly as percentage (ANOVA one-way test; *P* < 0.0001). The CD4/CD8 ratio was higher in HESNs compared with the more immunosuppressed UHIV CD4 <500 group, while the UHIV CD4 >500 group and ECs behaved similarly (ANOVA one-way test; *P* < 0.0001) (Table 1). Of note, patients under lipid-lowering treatment accounted for a minor 3.6% of the total patients studied (no data reported in HCs) and were similarly distributed between groups.

**TABLE 1.** Baseline characteristics of the study participants

Characteristic	UHIVs CD4 <500 (n = 28)	UHIVs CD4 >500 (n = 16)	ECs*^a^* (n = 8)	HESNs (n = 32)	HCs (n = 14)	n	*^b^*
Age, years*^c^*	37 (30–41)	36 (32–39)	39 (28–55)	36 (32–42)	33 (26–40)	97	ns (0.677)
Male/female, n male (%)	25/2 (93)	16/0 (100)	5/3 (63)	27/5 (84)	5/9 (36)	97	ns (0.161)
MSM*^d^*/other, n MSM (%)	23/4 (85)	14/2 (88)	6/2 (75)	27/5 (84)	N/D	83	ns (0.956)
Lipid-lowering treatment, n (%)	1 (4)	1 (6)	0	1 (3)	N/D	83	N/A
Virology/Immunology							
Plasma VL (log_10_)*^c^*	4.7 (3.9–5.4)	4.0 (3.2–4.5)	1.6 (1.6–1.9)	N/A	N/A	51	***
CD4+ T-cell count (cells/ml)*^c^*	306 (145–373)	701 (572–874)	549 (489–642)	N/D	N/D	51	***
CD4+ T-cell (%)	17 (11–26)	36 (28–40)	30 (27–37)	36 (31–44)	N/D	82	***
CD8+ T-cell count (cells/ml)*^c^*	822 (581–1,047)	1017 (709–1,112)	793 (601–1,052)	N/D	N/D	51	ns (0.276)
CD8+ T-cell (%)	58 (48–68)	44 (40–55)	42 (32–49)	22 (16–29)	N/D	82	***
Ratio CD4+/CD8+	0.26 (0.19–0.51)	0.78 (0.55–1.00)	0.76 (0.61–1.02)	1.55 (1.15–2.37)	N/D	83	***
Plasma lipids and lipoproteins							
TC (mg/dl)	167.5 ± 32.5	162.9 ± 25.0	208.8 ± 51.0	191.4 ± 37.2	175.7 ± 26.6	98	0.0031**
Triglycerides (mg/dl)	149.0 ± 102.1	130.1 ± 89.8	137.9 ± 78.5	106.1 ± 58.3	84.8 ± 42.2	98	0.07 ns
HDL-C (mg/dl)	38.1 ± 10.5	42.6 ± 8.6	48.6 ± 12.1	50.9 ± 12.1	60.4 ± 11.7	98	<0.0001***
LDL-C (mg/dl)	103.4 ± 28.7	97.9 ± 21.8	134.2 ± 43.1	119.9 ± 31.0	98.4 ± 25.0	87*^e^*	0.0138*
ApoAI (mg/dl)	110.8 ± 15.6	111.0 ± 8.8	120.5 ± 21.0	128.3 ± 16.4	132.6 ± 13.7	98	<0.0001***
ApoB (mg/dl)	82.6 ± 21.4	80.9 ± 13.9	103.1 ± 22.1	89.8 ± 20.4	87.6 ± 23.0	98	0.087 ns
ApoB/ApoAI	0.76 ± 0.21	0.73 ± 0.13	0.86 ± 0.14	0.71 ± 0.16	0.66 ± 0.19	98	0.1114 ns
Plasma inflammatory markers							
Lp(a) (log_10_)	1.22 ± 0.52	1.10 ± 0.38	1.12 ± 0.40	1.29 ± 0.49	1.05 ± 0.51	98	0.536 ns
hsCRP	0.45 ± 0.49	0.36 ± 0.80	0.32 ± 0.29	0.20 ± 0.31	0.14 ± 0.14	98	0.073 ns
Plasma functional assays							
CE	0.58 ± 0.13	0.74 ± 0.12	0.88 ± 0.25	0.78 ± 0.14	0.90 ± 0.13	98	<0.0001***
CE/HDL (×1,000)	15.70 ± 3.10	17.88 ± 3.88	19.14 ± 8.15	15.98 ± 4.45	15.11 ± 1.57	98	0.100 ns
CE/ApoAI (×1000)	5.25 ± 1.04	6.69 ± 1.16	7.42 ± 2.30	6.16 ± 1.37	6.82 ± 0.95	98	<0.0001***

Data are shown as mean ± standard deviation unless otherwise indicated. MSM, men who had sex with men; N/D, not determined; N/A, not applicable; ns, nonsignificant.

a One patient included in the EC group is a viremic controller (VL = 6,000 copies/ml).

b Statistical significance and *P* from χ^2^ or ANOVA one-way test comparing all groups (*P < 0.05; **P < 0.01; ***P < 0.0001).

c Data are shown as median (IQR, 25th to75th percentile).

d MSM include bisexual and homosexual.

e LDL-C measurements of 11 individuals could not be calculated.

### Metabolic alterations of the HIV-related groups

**Fig. 1.**
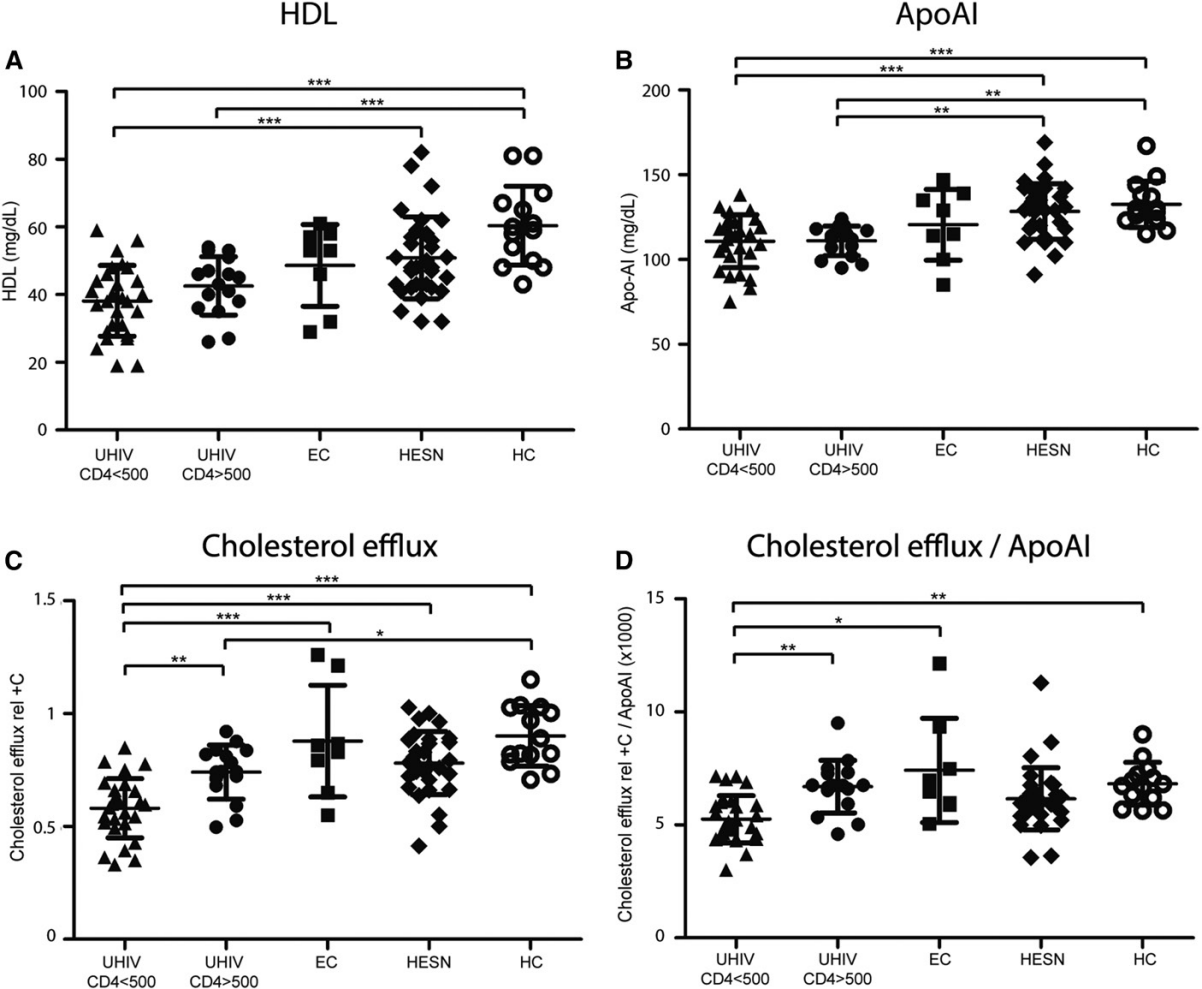
HDL-C content, apolipoprotein levels, and CE in patients at different stages linked to HIV infection (n = 97). A: Plasma HDL-C levels. B: Plasma ApoAI levels. C: CE capacity of ABDP samples normalized to the CE of a pool of sera from healthy donors (rel+C). D: CE capacity (rel+C) adjusted for ApoAI levels (ratio CE rel+C/ApoAI). ***P < 0.001; **P < 0.01; *P < 0.05.

The plasma analytics showed differences between the groups in terms of LDL-C (ANOVA one-way; *P* < 0.0138), HDL-C (*P* < 0.0001), and ApoAI (*P* < 0.0001) levels (Table 1). Groups showed similar LDL-C levels, as no significance was found in a one-to-one comparison posttest. HDL-C and its main lipoprotein component, ApoAI, were significantly lower in patients with detectable VL (UHIVs) compared with uninfected individuals (HESNs and HCs; Table 1; **Fig. 1A**, B). Interestingly, ECs situate between the UHIV CD4 >500 and HESN groups in terms of HDL-C (*t*-test; *P* = 0.20 and *P* = 0.64, respectively) and ApoAI levels (*t*-test; *P* = 0.26 and *P* = 0.13, respectively). hsCRP levels were the highest in UHIV CD4 >500 group (0.45 ± 0.49) and the lowest in HCs (0.14 ± 0.14; ANOVA one-way; *P* = 0.07), while no evidence of association between Lp(a) was found among the groups (ANOVA one-way; *P* = 0.53).

In summary, the quantitative levels of ApoAI and HDL-C were the most altered metabolic components in those groups linked to HIV infection. To further search on this lipid alteration, the qualitative status of the ApoAI in HDLs through a CE capacity assay was evaluated.

### HIV infection dampens CE

CE was measured in triplicate from patients’ ABDP and values expressed as normalized to the CE of a pool of sera from healthy donors (rel+C). CE was substantially altered between the groups and followed the same pattern as HDL-C and ApoAI levels. Diminished CE was observed in patients with detectable VL. However, higher CE was observed in patients with undetectable VL or the uninfected (both HESNs and HCs; Table 1; Fig. 1C). The UHIV CD4 <500 group presented significantly lower CE compared with the UHIV CD4 >500 group and to the rest of the groups (Bonferroni’s multiple test; *P* < 0.01 and *P* < 0.0001, respectively; Fig. 1C). The differential CE levels between the UHIV CD4 <500 group and the UHIV CD4 >500 group or HCs were also present after normalizing for ApoAI levels (Dunn’s multiple test; *P* < 0.01 in both cases; Fig. 1D). ECs showed a heterogeneous CE level; however, the average CE was similar to that of HCs. Strikingly, HESNs showed slightly lower CE and CE/ApoAI levels than HCs in the multiple comparisons; for this reason, this special group will be studied later on.

Overall, CE was sensitive to different stages of HIV progression or conditions linked to HIV (including HESNs and HCs), independently from ApoAI levels (Figs. 1D, 3D). Additionally, lower CE levels partially correlated with the inflammatory marker, hsCRP (Spearman’s *r* = −0.28; 95% interval −0.45 to −0.07; *P* = 0.007; n = 97), consistent with a lower degree of inflammation in those patients with higher CE.

### CE is associated to CD4+ T-cell count in UHIVs

We evaluated the associations of CE levels with the markers of disease progression in HIV+ patients in order to assess the role of CE in viral replication and immune response. For this assessment, we focused on the UHIV groups (CD4 <500 and CD4 >500) and evaluated whether CE is sensitive to CD4+ T-cell counts and HIV-1 VL. A linear regression model was estimated considering Ln (CE) as dependent variable, CD4+ T-cell count and log_10_ VL as independent variables, and age as a confounder.

**Fig. 2.**
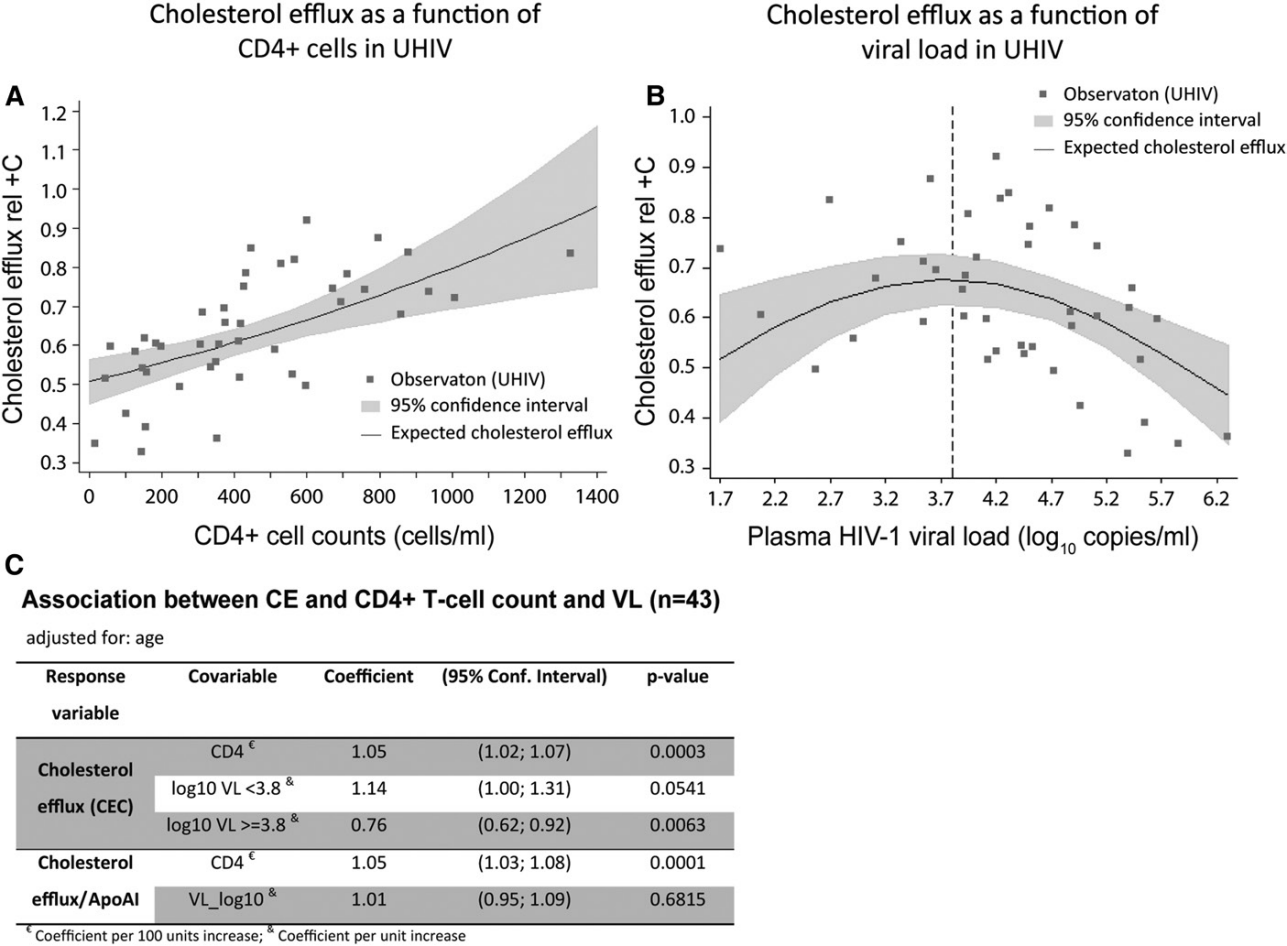
Statistical models of CE and CE/ApoAI in UHIVs (n = 43) adjusted for age. A: Scatterplot and linear regression model of the expected CE level with a 95% CI as a function of CD4+ T-cell counts. B: Scatterplot and regression model of the mean of CE with a 95% CI as a function of plasma HIV-1 VL. The regression model has the shape of a convex parabola and the dashed line indicates the position of the knot (approximately the vertex) used in the piecewise analysis. C: Piecewise regression models of CE and linear regression models of CE relative to ApoAI (CE/ApoAI) evaluated against VL and CD4+ T-cell counts. Only multivariate models are shown.

The results showed that both CD4+ T-cells and VL were significantly associated with CE in our model. CD4+ T-cell counts positively related with CE (regression model coefficient: 1.05; 95% CI: 1.02; 1.07; **Fig. 2A**), while VL showed a quadratic relationship (regression model coefficients, linear term = 1.61; 95% CI: 1.14; 2.27; quadratic term = 0.94; 95% CI: 0.90; 0.98; Fig. 2B). In terms of percentage change, per every 100 CD4+ T-cell increment, we expect to see about a 5% increase in CE. The effect of VL was positive at the beginning of its distribution, progressively decreases until the parabola vertex (3.8, approximately), and then turns negative. Because the subgroup analyzed was UHIVs, very few individuals were expected to present low VL values and, accordingly, the majority of individuals had VL values greater than 1,500/2,000 copies/ml. Hence, we adjusted a piecewise linear function for log_10_ VL with a knot at 3.8 VL log_10_ (vertex) to partition the functional form. The ascendant part had a small number of patients and the result was marginally significant (regression model coefficient: 1.14; 95% CI: 1.00; 1.31); the descendant part included most of the patients and was statistically significant (regression model coefficient: 0.76; 95% CI: 0.62; 0.92). These results confirm that VL had a significantly negative association with CE for VL >3.8 log_10_. Overall, in the UHIV group, CE is positively associated to CD4+ T-cell counts and negatively associated to VL for levels of HIV RNA >6,300 cp/ml.

CE is expected to increase with ApoAI levels because this is the main acceptor found in HDL particles, but CE values may reflect alterations in the functionality of HDLs or ApoAI, which should be independent from their quantitative levels. Therefore, a useful response variable to analyze HDL functionality, apart from CE, may be CE/ApoAI. CD4+ T-cell count presented a significant association with CE/ApoAI (regression model coefficient; 1.05; 95% CI: 1.03; 1.08), while there was no evidence of association between CE/ApoAI and VL (Fig. 2C). The interpretation of the model in terms of percentage change is that for 100 cell increments in CD4+ T-cells, we expect to see about a 5% increase of CE/ApoAI. This suggests that the correlation of CE with CD4+ T-cells is independent from the ApoAI levels, while there is no evidence of association with VL after adjusting CE for ApoAI (VL may affect ApoAI levels and consequently alter CE). To sum up, the HDL functionality measured as CE and adjusted for ApoAI levels is associated with the CD4+ T-cell count, but was not associated with VL in our sample of UHIVs.

Given that VL is negatively related to CD4+ T-cell counts in the context of HIV infection, we considered two hypotheses to further validate the model: *i*) CE capacity is impaired due to low levels of CD4+ T-cell-related immunological state; or *ii*) the presence of VL in blood reduces CE. To this aim, we evaluated three settings (n = 16 in each scenario; supplemental Table SII).

First, the effect of actively replicating viral particles (UHIVs) was evaluated toward a situation with basal replicating activity of the virus (ECs). Metabolic and inflammatory parameters and CE were compared. Main differences were observed in ApoB levels and in the subsequent ApoB/ApoA ratio, but CE remained unchanged. A second scenario analyzed UHIV patients matched for CD4+ T-cell counts, which differed from 1.6 logarithm of VL (*P* < 0.0001). No significant differences were found in any of the parameters evaluated, including CE. In a third scenario, two groups with similar VL, but differing in CD4+ T-cell counts, were compared. We confirmed an effective pairing per VL and a significant difference in CD4+ T-cells (*P* < 0.001). Moreover, CE showed a tendency to lower values in the group with lower CD4+ T-cells (0.58 ± 0.13 versus 0.76 ± 0.12; *P* = 0.054), and a significant difference in CE adjusted for ApoAI (*P* = 0.0027; supplemental Table SII). Thus, VL itself could not explain differences in CE, but CD4+ T-cell changes were associated with impaired CE in patients with similar VL.

### HESNs present altered CE capacity

**Fig. 3.**
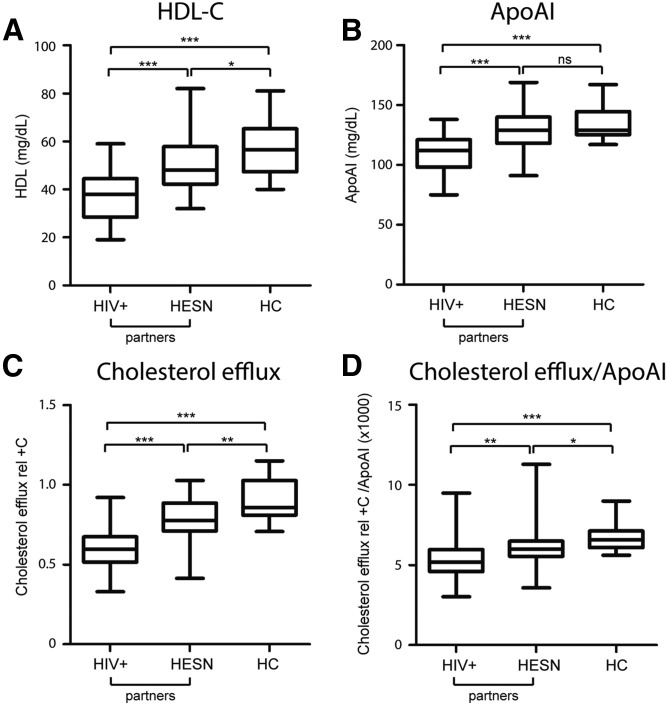
HDL-C content, apoAI levels, and CE in HESNs (n = 32), their HIV+ partners (n = 32), and HCs (n = 14). A: Plasma HDL-C levels. B: Plasma ApoAI levels. C: CE capacity of ABDP samples. D: CE capacity adjusted for ApoAI levels (ratio CE/ApoAI).

HDL-C, ApoAI, and CE levels in HESNs were compared with their respective HIV+ partners and to HCs. As expected, the seropositive individuals displayed significant differences in HDL-C, ApoAI, and CE compared with HESNs (paired *t*-test; *P* < 0.0001) and HCs (*t*-test; *P* < 0.0001; **Fig. 3A**–C). Strikingly, we observe differences in lipid profiles at a quantitative level between HESNs and HCs. HESNs showed significantly increased TC and LDL-C, but decreased HDL-C compared with HCs (Fig. 3A; supplemental Table SIII). The alteration in TC and LDL-C observed in HESNs follows an inverse tendency to that observed in HIV+ patients, while the decrease in HDL-C is a common feature of HESNs with HIV+ individuals compared with HCs.

In addition, HESNs showed significantly decreased CE (0.78 ± 0.03; n = 31) compared with HCs (0.90 ± 0.04; *t*-test; *P* = 0.012; Fig. 3C), also after normalizing for ApoAI (Mann Whitney test; *P* = 0.012; Fig. 3D; supplemental Table SIII), but no significant differences were present in ApoAI level between the two uninfected groups (*t*-test; *P* = 0.40; Fig. 3B; supplemental Table SIII). Overall, this indicates that diminished quantitative values of ApoAI occurred mainly in actively HIV+ patients, but impairment of CE appeared already in HESNs, which could indicate that qualitative CE is a more sensitive parameter than ApoAI levels.

## DISCUSSION

In this study, we found main differential plasma lipid alterations at the level of TC, HDL-C, and ApoAI between groups, and those were markedly altered in the most immunocompromised UHIVs compared with the noninfected groups of patients.

### CE in UHIVs

CE was sensitive to the different groups related to HIV infection and significantly reduced in UHIV patients compared with HCs, particularly those with lower CD4+ T-cell counts. CE was also decreased after adjusting for ApoAI level. CE adjusted for HDL-C level showed the same tendency as when adjusted for ApoAI level, while the statistical comparison result was nonsignificant; this may be explained because ApoAI is the actual acceptor of cholesterol, while circulating HDL particles are heterogeneous and can already be loaded (saturated) with cholesterol, thus with a reduced number of functional acceptors.

A previous study reported no differences in CE or ApoAI levels in UHIV patients compared with healthy individuals (17), but, consistent with our results, in a recent and exhaustive study, El Khoury et al. (15) showed that CE is diminished in UHIVs. We hypothesized that high VL or immunosuppression secondary to viral replication (CD4+ cell counts) may directly or indirectly explain the reduction of CE capacity.

### CE associated with CD4+ T-cell counts

Our study focused on ART-naïve individuals, and we found a gradient of CE levels dependent on both CD4+ T-cell counts and HIV-1 VL. Of note, a positive correlation of CE with CD4+ T-cell counts and a negative relation with plasma VL had already been pointed out (15); but because these two variables strongly interrelate, the contribution and thresholds of either VL or CD4+ T-cell status on CE had not been assessed. Our experimental setting allowed us to analyze individually the role of VL and immune status (assessed by CD4+ and CD8+ T-cell counts) on CE. We found a major association of CE with both CD4+ T-cell counts and VL in UHIV patients. CE correlated positively with CD4+ T-cell counts, while VL was negatively related from values of VL >3.8 log_10_. However, only the CD4+ T-cell count was associated to CE after adjusting for ApoAI levels.

Our data suggest that VL may alter CE, mainly by modifying the levels of ApoAI. Consistently, HIV VL is associated with various lipid parameters (21), suggesting a direct effect of viral replication on lipid levels (and consequently on net CE). CD4+ T-cell count in HIV+ patients has been correlated with ApoAI levels (17) and HDL-C (21). In this study, we found that, in considering a group of UHIV patients with equal ApoAI levels (variable CE/ApoAI), lower CE was associated with lower CD4+ T-cell count, secondary to HIV infection.

### Lipid profile and CE in HESNs

HESN individuals are of great interest for HIV vaccine development and as a model of immune response to HIV. Strikingly, HESN individuals showed higher TC and LDL-C, contrary to UHIV patients and in line with a study performed in children that were HIV-exposed during fetal development and their mothers were on ART (22). Our HESN group showed lower HDL-C and HDL functionality compared with HC individuals, also after adjusting for ApoAI levels. This is the first time, to our knowledge, that CE is evaluated in HESNs, and we suggest that exposure to HIV may be enough to impair HDL functionality with minimal and nonsignificant diminution in ApoAI levels. Therefore, HESNs may also help for the study of the initial steps of reverse cholesterol transport in HIV individuals. Whether a specific lipid profile (e.g., higher TC and LDL-C and lower HDL-C) or lipoprotein profile is protective to HIV infection will need further studies.

Regarding the role of immune activation in HESNs, some data support that immune activation favors protection from HIV infection (23, 24). Other authors justify the decreased susceptibility to infection with a reduced inflammatory response in HESN individuals (25, 26). Cholesterol metabolism may be decreased by immune activation or by certain specific immune responses to HIV (26), which occur in HESNs.

### Limitations of the study and conclusions

One of the main limitations of this study is the relatively small number of individuals included in each group, particularly ECs (n = 8), which additionally showed a heterogeneous distribution in HDL-C levels and consequent CE. More than 80% of individuals were men. Of note, the proportion of women was higher in the HC group compared with the other groups, thus a sex bias ratio has to be taken into account when comparing the HC (36% men) and the HESN (84% men) groups. Although storage of plasma at −80°C is a common practice, a recent study concluded the need of cryoprotectants to preserve CE in purified HDLs (27). On the other hand, the main strength of this study is that five groups with diverse HIV-related conditions were evaluated; serodiscordant couples could be studied pairwise toward their own partners; lipid-lowering agent consumption was reported for all groups, except for HCs, and only three individuals receiving lipid-lowering agents from different groups were reported. Smoking was not registered.

Changes in lipid species, mainly carried by plasma HDLs, LDLs, and VLDLs, may contribute to the inflammatory setting in the context of HIV (28). Inflammation has been shown to remodel the HDL proteome and lipidome and subsequently alter CE (29, 30). Here, we observed a partial correlation of CE with the inflammatory marker, hsCRP. An ART-related improvement of CE was recently related to reduced immune activation (by a change in the percentage of classical vs. nonclassical monocytes) (16). It is thus possible that the immune activation caused by HIV-infection not only alters HDL-C levels but also the profile of lipid species in HDL particles. It could turn down their function capacity here assessed as CE.

In summary, our experiments corroborate that CE is associated with HIV viral replication and CD4+ T-cell counts. CE is markedly dampened in UHIV patients, particularly those with CD4 <500. For the first time, this study shows that CE is impaired in HESNs compared with HCs. We found a positive association of CE with CD4+ T-cell count, secondary to the uncontrolled HIV-1-infection, even after adjusting for ApoAI levels.

## Supplementary Material

Supplemental Data
