# Supplementary material for: Cholesterol efflux responds to viral load and CD4 counts in HIV+ patients and is dampened in HIV exposed
Source: J Lipid Res. 2018 Sep 13;59(11):2108–15. doi: 10.1194/jlr.M088153 (PMC6210904; doi:10.1194/jlr.M088153)
Supplement: Supplemental Data [file supp_59_11_2108__index.html]

Cholesterol efflux responds to viral load and CD4 counts in HIV+ patients and is dampened in HIV exposed — Supplemental Data 

# Cholesterol efflux responds to viral load and CD4 counts in HIV+ patients and is dampened in HIV exposed

## Supplemental Data

- Supplementary methods
- Supplementary figure 1
- Supplementary Table 1
- Supplementary Table 2
- Supplementary Table 3
