# Supplementary material for: Cholesterol efflux responds to viral load and CD4 counts in HIV+ patients and is dampened in HIV exposed
Source: J Lipid Res. 2018 Sep 13;59(11):2108–15. doi: 10.1194/jlr.M088153 (PMC6210904; doi:10.1194/jlr.M088153)
Supplement: Supplemental Data [file supp_M088153_OTort_Supplementary_methods.pdf]

Supplementary methods

Protein extraction and Inmunoblot

Human monocyte THP-1 cells were cultured in RPMI 1640 with L-Glutamine (Lonza, Verviers, Belgium, #12-702F) supplemented with 10% heat-inactivated fetal bovine serum (FBS, Gibco, #10270-106), 100 U/ml penicillin, 100 mg/ml Penicillin-Streptomycin (Lonza, Verviers, Belgium, #DE17-602E). Cells were maintained in a humidified atmosphere with 5% CO_2 at 37ºC. THP-1 monocytes were differentiated into a macrophage-like cell line in the presence of 100 nM phorbol myristoyl acetate (PMA, Sigma #79346) for 48h. ABCA1 protein expression was determined in THP-1 cells by immunoblotting of total protein extracts after lysis in a buffer containing 50 mM Tris-HCl pH 7.4, 0.1% NP40, 0.1% SDS (BDH), 0.1% DeoxycholicAcid, 0.1 mM EDTA, 0.1 mM EGTA, 5.35 mM sodium fluoride, 2.76 mM Sodium pyrophosphate freshly supplemented with 1mM Sodium orthovanadate, 1mg/ml protease inhibitor (Roche) and 0.25 mg/ml Pefabloc (AEBSF). Cell lysates where quantified with the Nanodrop and equal amounts of total protein were loaded in a 8-16 % SDS-poliacrilamyde gel electrophoresis (GE Healthcare Amsterdam, #28-9898-07).

Proteins were transferred onto a polyvinylidene fluoride (PVDF) membrane in a TransBlot SD semi-dry (BioRad) system and blocked in 5 % nonfat milk in TBST (20 mmol/L Tris base pH 7.6, 150 mM NaCl, 0.05 % Tween-20) before incubation with primary antibodies: anti-ABCA1 (1:2500; Millipore, #MAB10005) and anti-β-actin (1:1000; BD, #612656) in TBST 2 % nonfat milk, overnight (O/N) shaking at 4 ºC. Membranes were washed, incubated with secondary antibody HRP Goat anti-Mouse Ig (1,5000; BD, #554002) and chemoluminiscent signal was acquired using Clarity Western ECL Substrate (BioRad, #1705060) and ImageQuant LAS500 (GE Healthcare).

Cholesterol efflux method validation

Methods to assess cholesterol efflux (CE) capacity have been previously described and accurately validated (3, 31), mainly using mouse macrophage cell lines. Given the cell-base dependence of this assay, we first tested the validity and accuracy of the modifications we included to the existent protocol. A human monocyte/macrophages cell line, THP-1, was selected, as the plasma samples to be tested were also human and esterified cholesterol is low in THP-1 cells compared to murine cell lines (19). CE is highly dependent on the expression of ABCA1 cholesterol receptor, thus we corroborate that ABCA1 was induced in THP-1 cells after PMA differentiation (Supplementary Figure 1A). We found linear CE in the range of 0.5 to 2% ABDS, based on pooled plasma from healthy donors, similar to previous reports (32) (Supplementary Figure 1B).

Six plasma samples were analyzed for lipid and lipoprotein parameters before and after ApoB-depletion treatment in order to evaluate the ApoAI/HDL-C recovery and the efficiency of the depletion process. Recovered ApoAI and HDL-C levels were 69% and 52%, respectively, while total cholesterol accounted for 18% and triglycerides for 12% after depletion. Remaining LDL levels were 8% in average after ApoB-depletion of the plasma sample. CE was measured in triplicates for each sample and the assay was divided in six batches of 4-5 plates per batch. We additionally used a multichanel electronic pipette (1200 Xplorer plus 8 channel, Eppendorf, #Z767239) and high precision filter tips (ep Duafilter T.I.P.S 50-1250 ul, Eppendorf, #022494002) to minimize pipetting errors. As result, the correlation between replicates was excellent (Pearson's $r=93$; $p<0.0001$; $n=96$; Supplementary Figure 1C).

CE values of individual patients were expressed as normalized by CE of a serum pool that was freshly prepared in each batch and run in parallel with the patient sample. Real cholesterol efflux measurements showed almost an exact linear relationship with real cholesterol efflux relative to the positive control (Pearson's $r=0.92$; $p<0.0001$; $n=100$; Supplementary Figure 1D), indicating very low inter-batch variability.

Cholesterol efflux assay

THP-1 monocytes (0.4x10^6 cells) were seeded in a 24-well plate and derived into macrophage-like cells as already described. After washing with phosphate-buffered saline (PBS), the cells were treated with 10% FBS RPMI media containing 1 μCi/ml (18.87 nM) of [1,2-^{3}H] cholesterol (Perkin Elmer, #NET139001MC) for 24 hours at 37 ºC and 5 % CO_2 at the radioactivity biohazard laboratory. Cells were washed twice with PBS and incubated 18-20h with equilibration media (EM), composed by RPMI supplemented with 100 mg/ml Penicillin-Streptomycin and 1 % bovine serum albumin (BSA, Sigma #A7906-100G). Cells were then washed with PBS and 2% of the ABDP to be tested (cholesterol acceptor) was applied to the cells in EM. A 2% ABDS from a pool of healthy donors (internal positive standard control) dissolved in EM and EM alone (negative control) were included per triplicate in each plate. After 18-20h incubation with the cholesterol acceptors, media containing the effluxed radiolabeled cholesterol was gathered and centrifuged (13,200 rpm; 10min at room temperature) to remove unattached cells and aggregates. Cells were washed in PBS and subsequently disrupted in milliQ water at -20 ºC for at least 2h. 0.1 ml fraction (1/5 of the total volume) of both supernatants and cells extracts containing intracellular radiolabeled cholesterol, were separately measured by liquid scintillation counting. Radioactivity was measured in excess scintillation liquid (OptiPhase HiSafe 3, Perkin Elmer, #1200-437) in an automatic TDCR liquid scintillation counter (Hidex 300 SL).

Cholesterol efflux capacity was calculated per triplicate as [cpm media / (cpm media + cpm cells (X)) x 100]. Real cholesterol efflux (rCE) expressed as [cholesterol efflux – negative control cholesterol efflux (no HDL)] corrects for plasma unrelated cholesterol efflux. Real cholesterol efflux relative to the positive control (rCE/C+) was calculated as [real cholesterol efflux / positive control real cholesterol efflux (pool HDL)] in order to correct for inter-batch variability.
