# Supplementary material for: Cholesterol efflux responds to viral load and CD4 counts in HIV+ patients and is dampened in HIV exposed
Source: J Lipid Res. 2018 Sep 13;59(11):2108–15. doi: 10.1194/jlr.M088153 (PMC6210904; doi:10.1194/jlr.M088153)
Supplement: Supplemental Data [file supp_M088153_OTort_Sup_fig1_CEvalidation.pdf]

Supplementary Figure

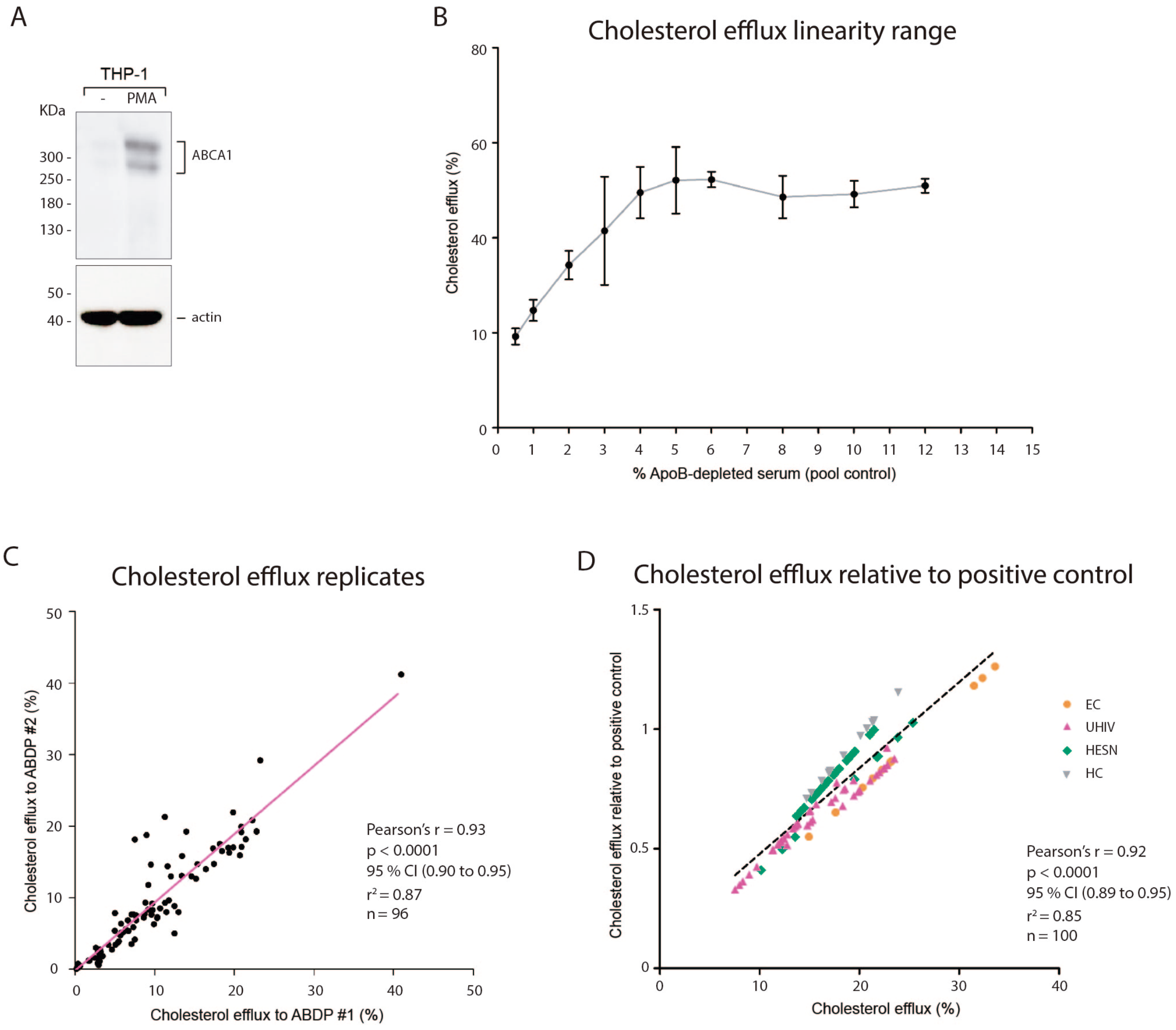

Supplementary figure 1:

Cholesterol efflux (CE) capacity assay optimization in THP-1 cells using ApoB-depleted plasma (ABDP). A) Expression of ATP-binding cassette transporter (ABCA1), a main ApoAI-dependent cholesterol transporter, after THP-1 differentiation with 100 nM PMA during 48 h. B) The linearity range of the assay was determined measuring CE along increasing percentages of ABDS (pool of healthy donors) incubated with the cells. The range between 0.5-2% ABDP fitted with the highest linearity, therefore 2% ABDP was selected for the assays along this work. C) Reproducibility of the

CE measurements assessed by confronting duplicate #1 with duplicate #2 of the ABDP sample. High level of correlation (r^2= 0.87; $p<0.0001$) was observed. D) In order to reduce inter-batch variability, CE was relativized by an internal positive control (pool of ABDP from healthy donors). CE highly correlated (r^2= 0.85; $p<0.0001$) with CE from the positive control.
