# Supplementary material for: Cholesterol efflux responds to viral load and CD4 counts in HIV+ patients and is dampened in HIV exposed
Source: J Lipid Res. 2018 Sep 13;59(11):2108–15. doi: 10.1194/jlr.M088153 (PMC6210904; doi:10.1194/jlr.M088153)
Supplement: Supplemental Data [file supp_M088153_OTort_Sup_TableI_CE_validation.pdf]

Supplementary Table I. Cholesterol efflux assay validation

Batch	n plates/batch	ABDP	Cholesterol efflux C-	Real cholesterol efflux C+	n patients	replicates/n	intra-assay CV$ (between triplicates)	intra-batch CV# (inter-plate)
**1**	4	PL	7.4	22.8	24	triplicates	6.3 (0.8-11.7)	7.9 (7.9-8.0)
**2**	4	PL	8.6	21.5	24	triplicates	6.5 (0.9-15.6)	6.1 (4.8-7.4)
**3**	4	PL	8.0	24.7	24	triplicates	4.5 (0.9-12.9)	7.9 (3.4-12.3)
**4**	4	PL	7.4	26.2	24	triplicates	5.5 (1.2-10.6)	5.8 (4.0-7.5)
**5**	5	PL	7.3	20.7	28	triplicates	4.6 (1.4-8.4)	7.0 (5.4-8.6)
**Total Study**	**21**	**PL**	**7.7±0.6&**	**23.2±2.3&**	**124**	**triplicates**	**5.5 (4.5-6.5)**	**6.9 (5.8-9.8)χ**

ABDS/P, ApoB-depleted serum/plasma; PL, plasma; C-, negative control (no ABDP); positive control (ABDS from a pool of human plasma); CV, coefficient of variation; $ Intra-assay CV is calculated as the average CV between triplicate values of cholesterol efflux of the same patient. # Intra-batch CV is determined as the CV of cholesterol efflux of the negative and positive control between plates in a batch. & Mean ± standard deviation. χ Inter-batch CV assess the dispersion between positive control real cholesterol efflux values (column *Real cholesterol efflux C+)* among batches.
