# Supplementary material for: Cholesterol efflux responds to viral load and CD4 counts in HIV+ patients and is dampened in HIV exposed
Source: J Lipid Res. 2018 Sep 13;59(11):2108–15. doi: 10.1194/jlr.M088153 (PMC6210904; doi:10.1194/jlr.M088153)
Supplement: Supplemental Data [file supp_M088153_OTort_Sup_TableII_Charact_StudyParticipants.pdf]

Supplementary Table II. Characteristics of the study participants in three scenarios

	Scenario 1				Scenario 2				Scenario 3			
	UHIV *versus* EC (match for CD4)				UHIV *versus* UHIV (match for CD4)				UHIV *versus* UHIV (match for VL)			
**Participant characteristics**	EC (n= 8)	UHIV, CD4 match 1:1 (n= 8)	n	p-value[£]	UHIV low VL (n= 8)	UHIV high VL, CD4 matched 1:1 CD4 (n= 8)	n	p-value[£]	UHIV high CD4 (n= 8)	UHIV low CD4, VL matched 1:1 (n= 8)	n	p-value[£]
Age, years[$]	39 (28-55)	36 (33-40)	16	ns (0.290)	38 (35-43)	34 (29-40)	16	ns (0.175)	36 (31-37)	33 (29-38)	16	ns (0.60)
Male/Female, n male (%)	5/3 (63)	8/0 (100)	16	N/A	8/0 (100)	7/1 (88)	16	N/A	8/0 (100)	7/1 (88)	16	N/A
MSM[&]/Other, n MSM (%)	6/2 (75)	8/0 (100)	16	N/A	7/1 (88)	7/1 (88)	16	ns (1)	7/1 (88)	6/2 (75)	16	ns (1)
Plasma viral load (log10)[$]	1.57 (1.57-1.86)	4.3 (4.0-4.6)	16	** (0.0078)	3.0 (2.6-3.5)	4.6 (4.1-5.4)	16	** (0.0002)	4.33	4.29	16	ns (0.283)
CD4+ T-cell count (cells/ml)[$]	549 (489-642)	545 (461-653)	16	ns (0.195)	510 (355-842)	516 (357-849)	16	ns (0.377)	734 (618-858)	267 (149-404)	16	** (0.001)
CD8+ T-cell count (cells/ml)[$]	793 (601-1052)	960 (790-1031)	16	ns (0.641)	616 (535-1035)	1079 (858-1315)	16	ns (0.383)	1052 (859-1156)	851 (516-1364)	16	ns (0.148)
Ratio[$] CD4+/CD8+	0.76 (0.61-1.02)	0.57 (0.52-0.82)	16	ns (0.273)	0.78 (0.42-1.18)	0.51 (0.26-0.84)	16	ns (0.155)	0.78 (0.54-0.92)	0.24 (0.20-0.49)	16	ns (0.109)
**Biochemistry[¥]:**												
Total cholesterol (mg/dL)	208.8±51.0	159.9±26.1	16	ns (0.090)	183.5±38.1	172.0±42.3	16	ns (0.667)	162.4±27.3	163.3±18.4	16	ns (0.947
Triglycerides (mg/dL)	137.9±78.5	167.8±161.4	16	ns (1.000)	111.1±33.6	189.0±117.8	16	ns (0.383)	158.4±111.5	108.4±77.8	16	Ns (0.25)
HDL-C (mg/dL)	48.6±12.1	44.1±10.1	16	ns (0.440)	42.8±5.7	41.4±11.2	16	ns (0.712)	40.3±10.1	41.5±9.1	16	Ns (0.82)
LDL-C (mg/dL)	134.2±43.1	87.4±18.5	12	ns	118.5±32.4	101.0±32.1	15	ns	96.3±24.1	102.1±18.4	12	ns

				(0.313)				(0.937)				(0.927)
ApoAI (mg/dL)	120.5±21.0	110.0±9.7	16	ns (0.256)	113.4±7.9	113.0±17.7	16	ns (0.960)	108.6±10.7	114.4±11.2	16	ns (0.335)
ApoB (mg/dL)	103.1±22.1	71.9±13.0	16	* (0.025)	94.8±19.1	83.9±19.3	16	ns (0.378)	40.3±10.1	41.5±9.1	16	ns (0.816)
ApoB/ApoAI	0.86±0.14	0.66±0.16	16	* (0.034)	0.83±0.14	0.75±0.15	16	ns (0.275)	0.74±0.14	0.73±0.18	16	ns (0.922)
Lp(a) (log10)	1.18±0.40	0.90±0.33	16	ns (0.250)	1.42±0.45	1.08±0.46	16	ns (0.114)	1.15±0.40	1.43±0.46	16	ns (0.243)
hsCRP	0.32±0.29	0.12±0.07	16	ns (0.148)	0.62±1.11	0.30±0.29	16	ns (1.00)	0.18±0.16	0.27±0.36	16	ns (0.945)
Cholesterol efflux	0.88±0.25	0.78±0.10	16	ns (0.361)	0.69±0.13	0.70±0.17	16	ns (0.814)	0.76±0.12	0.58±0.13	16	ns (0.054)
Cholesterol efflux/ApoAI (x1000)	7.41±2.30	7.16±1.25	16	ns (0.813)	6.10±1.24	6.29±1.69	16	ns (0.783)	7.12±1.22	5.17±1.13	16	* (0.027)

UHIV, untreated HIV infected patients; EC, elite controllers; Lp(a), lipoprotein(a); hsCRP; high-sensitive C-reactive protein; N/A, not applicable;

£Paired t-test (Wilcoxon signed rank test for non-parametric variables and Paired t-test for normal distribution); N/A, not applicable; ns, non-significant; * p< 0.05; ** p< 0.01; *** p<0.0001.

$Data shown median (IQR, 25th – 75th percentile);

¥ Data shown as mean ± standard deviation.

CD4+ T-cell count and VL had an effective pairing (***).
