# Supplementary material for: Cholesterol efflux responds to viral load and CD4 counts in HIV+ patients and is dampened in HIV exposed
Source: J Lipid Res. 2018 Sep 13;59(11):2108–15. doi: 10.1194/jlr.M088153 (PMC6210904; doi:10.1194/jlr.M088153)
Supplement: Supplemental Data [file supp_M088153_OTort_Sup_TableIII_HESNvsHC.pdf]

Supplementary Table III. Characteristics of HC and HESN

Participant characteristics	HC (n=14)	HESN (n= 32)	n	p-value[£]
Age, years[$]	33 (26-40)	36 (32-42)	46	ns (0.253)
Male/Female, n male (%)	5/9 (36)	27/5 (84)	46	* (0.028)
**Clinical data:**				
Presumed mode of HIV transmission/exposure, n (%):			46	N/A
MSM[&]	N/D	27 (84)		
Other	N/D	5 (16)		
**Biochemistry[¥]:**				
Total cholesterol (mg/dL)	175.7±26.6	191.4±37.2	46	* (0.161)
Triglycerides (mg/dL)	84.9±42.2	106.1±58.3	46	ns (0.252)
HDL-C (mg/dL)	60.4±11.7	50.9±12.1	46	* (0.017)
LDL-C (mg/dL)	98.4±25.0	119.9±30.9	43	* (0.029)
ApoAI (mg/dL)	132.6±13.7	128.3±16.4	46	ns (0.403)
ApoB (mg/dL)	87.6±23.0	89.8±20.4	46	ns (0.739)
ApoB/ApoAI	0.67±0.18	0.71±0.16	46	ns (0.457)
Lp(a) (log10)	1.05±0.51	1.29±0.49	46	ns (0.142)
hsCRP	0.14±0.14	0.20±0.31	46	ns (0.792)
Cholesterol efflux	0.90±0.13	0.78±0.14	46	** (0.009)
Cholesterol efflux/ApoAI (x1000)	6.82±0.95	6.16±1.37	46	* (0.023)

HC, healthy control individuals; HESN, HIV-exposed seronegative; Lp(a), lipoprotein(a); hsCRP; high-sensitive C-reactive protein; N/D, not determined; N/A, not applicable.

[£]T-test for normal distributed variables and Kruskal-Wallis test for non-parametric variables; ** $p<0.01$; * $p<0.05$.

[$]Data shown median (IQR, 25th – 75th percentile);

[¥] Data shown as mean ± standard deviation.
